# Development of a Machine Learning-Based Screening Method for Thyroid Nodules Classification by Solving the Imbalance Challenge in Thyroid Nodules Data

**DOI:** 10.34172/jrhs.2022.90

**Published:** 2022-08-29

**Authors:** Sajad Khodabandelu, Naser Ghaemian, Soraya Khafri, Mehdi Ezoji, Sara Khaleghi

**Affiliations:** ^1^Student Research Committee, School of Medicine, Faculty of Health, Babol University of Medical Science, Babol, Iran; ^2^Department of Radiology, Babol University of Medical Sciences, Babol, Iran; ^3^Research Center for Social Determinants of Health, Health Research Institute, Department of Biostatistics and Epidemiology, Faculty of Health, Babol University of Medical Sciences, Babol, Iran; ^4^Faculty of Electrical and Computer Engineering, Babol Noshirvani University of Technology, Babol, Iran

**Keywords:** Machine learning, Support vector machines, Thyroid nodule, Ultrasonography

## Abstract

**Background:** This study aims to show the impact of imbalanced data and the typical evaluation methods in developing and misleading assessments of machine learning-based models for preoperative thyroid nodules screening.

**Study design:** A retrospective study.

**Methods: **The ultrasonography features for 431 thyroid nodules cases were extracted from medical records of 313 patients in Babol, Iran. Since thyroid nodules are commonly benign, the relevant data are usually unbalanced in classes. It can lead to the bias of learning models toward the majority class. To solve it, a hybrid resampling method called the Smote-was used to creating balance data. Following that, the support vector classification (SVC) algorithm was trained by balance and unbalanced datasets as Models 2 and 3, respectively, in Python language programming. Their performance was then compared with the logistic regression model as Model 1 that fitted traditionally.

**Results:** The prevalence of malignant nodules was obtained at 14% (n = 61). In addition, 87% of the patients in this study were women. However, there was no difference in the prevalence of malignancy for gender. Furthermore, the accuracy, area under the curve, and geometric mean values were estimated at 92.1%, 93.2%, and 76.8% for Model 1, 91.3%, 93%, and 77.6% for Model 2, and finally, 91%, 92.6% and 84.2% for Model 3, respectively. Similarly, the results identified Micro calcification, Taller than wide shape, as well as lack of ISO and hyperechogenicity features as the most effective malignant variables.

**Conclusion: **Paying attention to data challenges, such as data imbalances, and using proper criteria measures can improve the performance of machine learning models for preoperative thyroid nodules screening.

## Background

 Thyroid nodules are frequent, and the majority of them are benign. This illness is responsible for 1% of all human malignancies.^1^ Its prevalence is around 50%-60% in the United States and 22.4% in Iran, representing a considerable increase over previous years. It is generally more prevalent in northern Iran, particularly in coastal and mountainous areas. The prevalence of thyroid nodules in the general population ranges from 19% to 68%, with 5%-10% of nodules being cancerous.^1^ Therefore, the primary goal of diagnosing thyroid nodules is to differentiate malignant nodules from benign ones. Fine needle aspiration (FNA) is the gold standard for diagnosing this disease. However, about 15%-30% of FNA results are indeterminate cytological diagnoses.^2,3^ To achieve a decisive interpretation in these cases, FNA is often repeated. Therefore, the risks that the patient poses due to repeated FNA (with potentially aggressive characteristics) for its uncertain result will also cause frustration and stress to the patient and impose additional medical costs.^4^ These problems are exacerbated when the patient's nodule is benign. A medical imaging-based screening approach utilized ahead of the FNA procedure can significantly assist thyroid nodule specialists. Using such a system alone is not sufficient for diagnosis; however, it substantially influences the diagnosis of remarkably benign thyroid nodules. Proper diagnosis of benign nodules reduces invasive FNA procedures for a wide range of healthy subjects, avoiding the potential side effects and expenses.

 In the last few decades, some artificial intelligence (AI) algorithms, especially deep-learning and machine learning algorithms, have been developed for classification and prediction.^5^ These algorithms have had acceptable results in most fields, compared to other traditional methods. Machine learning models can be one of the most suitable methods to replace conventional methods since they do not impose any basic assumptions on data distribution. Moreover, they do not charge any restrictions on the functional form of the relationship between independent and dependent variables.^6,7^

 This study pursued two main goals: the first was to examine the most widely used machine learning model in two ways that fitted with balanced and unbalanced data. The second was to investigate the impact of using the appropriate index to report the model's performance when encountering unbalanced data.

## Methods

###  Data

 This retrospective study was performed in Babol, Iran. The demographic and sonographic data for available patients were from patients' medical records between 2019 and 2020. Inclusion criteria were patients with a diagnosis of thyroid nodule by FNA indication, 6-month follow-up, cytological results reported by the pathologist, full consent to participate in the study, and no specific cysts. On the other hand, patients with benign cytology without a 6-month follow-up and those whose results were unavailable after the FNA procedure were excluded from the study. All information collected for patients in this study was diagnosed and recorded by a radiologist with more than 10 years of expertise.

 This study included two quantitative and nine categorized variables. For model development, the categorized variables were converted to dummy variables (A variable with n categories is transformed into n-1 binary variables.) so that in all of them, according to Table 1, the first category was considered a reference (the first category is marked with a star symbol). The name of each category was regarded as a variable name. Finally, 16 variables were prepared for model development.

**Table 1:** Descriptive information and the relationship between each of the variables in the study with the response variable (nodule type) based on Model 1(multiple logistic regression)

**Variables**	**Nodule’s type**		**Model 1**		**Bivariate tests**
	**Malignant**	**Benign**	**OR (CI 95%)**	***P*** ** value**	***P*** ** value**
**Continuous variables**	**Mean (SD)**	**Mean (SD)**			
Age	40.75 (13.63)	48.15 (12.00)	0.96 (0.93, 0.99)	0.034	0.001
Nodule size	14.80 (8.66)	20.65 (14.42)	0.99 (0.95, 1.03)	0.537	0.001
**Categorized variables**	**Number**	**Number**	**OR (CI 95%)**	***P*** ** value**	***P*** ** value**
Gender					0.788
Male	7	47	1.00		
Female	54	323	0.59 (0.20, 1.79)	0.355	
Location					0.184
Isthmus	1	13	1.00		
Right lobe	26	196	0.69 (0.07, 7.20)	0.761	
Left lobe	34	161	0.95 (0.09,9.90)	0.963	
Echogenicity					0.001
Marked hypo	15	4	1.00		
Hypo	35	96	0.40 (0.09, 1.87)	0.246	
Iso	11	265	0.05 (0.01, 0.27)	0.001	
Hyper	0	5	0.18 (0.02, 1.87)	0.999	
Margin					0.001
Smooth	46	365	1.00		
Irregular or micro lobulated	15	5	0.84 (0.20, 3.55)	0.814	
Calcification					0.001
No categorize	25	303	1.00		
Micro calcifications	34	41	9.61 (4.03, 22.95)	0.001	
Macro calcifications	2	26	1.06 (0.14, 7.90)	0.955	
Nodule Shape					0.001
Wider than tall	33	359	1.00		
Taller than wide	28	11	7.06 (2.34, 21.30)	0.001	
Composition					0.006
Solid	60	305	1.00		
Predominantly cystic	0	21	0.95 (0.17, 1.47)	0.998	
Predominantly solid	1	44	0.25 (0.03, 2.10)	0.201	
Vascularity					0.016
No	41	298	1.00		
Yes	20	71	2.76 (1.07, 7.15)	0.036	
Lymphadenopathy (lnp)					0.001
No	54	370	1.00		
Yes	7	0	4.2 (1.04, 10.56)	0.999	

 The data collected in this field have been unbalanced in malignant and benign classes because most thyroid nodules are benign. Imbalance data can lead to models being misled towards the majority class. Accordingly, a combination resampling method called Smote-Tomek was used to solve this problem in this study.^8-12^ Smote-Tomek was created from a combination of Smote and Tomek methods. Unlike the random sampling method, the Smote algorithm, to increase the sample size in the minority class, prefers to build or simulate a new sample (using the K-nearest neighbors algorithm) rather than copy the existing samples in the minority class.^13^ This advantage minimizes overestimation in the model results, and it is the cause of using this combined method to balance the data. Imbalanced-Learn Package in Python was used to perform balancing methods.^14^

###  Models

 Two classification methods were used, namely logistic regression (LR)^15^ and support vector machines (SVM).^16^ The reason for choosing the LR method is the widespread use and popularity of this statistical model for solving classification problems traditionally and also being one of the basic models of machine learning.^17^ Support vector machines called SVM are supervised learning algorithms that can be used for classification and regression problems as support vector classification (SVC) and support vector regression (SVR).^18^ SVC is a common type of classifier for high-dimensional data by constructing a multidimensional hyperplane to obtain the optimal solution for classification using statistical methods. Choosing the SVC is based on the most widely used statistical models for classifying thyroid problems that have a long history in this field.^19,20^ Moreover, the first commercialized thyroid US system using AI was utilized in this model.^21,22^

###  Model development

 In this study, three classification models were fitted in the following order:

*Model 1*: Multiple LR was fitted in the traditional way using SPSS software (version 25) and all data without cross-validation method.

*Model 2*: The SVC classifier uses original data (unbalance data) and the cross-validation method, randomly divided into two categories of training and testing to fit the model with a ratio of 70 to 30. Following that, training dataset was used to model learning, and the testing dataset was utilized to evaluate the model. It is worth mentioning that five random replications were used for cross-validation to prevent overfitting.^23^

*Model 3*: SVC model using balanced data.

 Model fitting steps of this model are similar to Model 2 with the difference that after dividing the dataset into training and testing, the training dataset was balanced using the Smote-Tomek algorithm and then used for model training. The process of Smote-Tomek is as follows:

1. (Start of Smote algorithm) For random sample x_i_ ∈ minority class, compute the k nearest neighbor’s Euclidean distance.
2. Select a neighbor x_j_ randomly from the k nearest neighbors of x_i_.
3. According to the following formula, it produces a new synthetic sample between x_i_ and x_j_: δ ∈ [0, 1] is a random parameter
4. Repeat steps 1-3 until the desired proportion of the minority class is met. (End of Smote)
5. (Start of Tomek-Links) Choose random sample xj from the majority class. Euclidean distance calculation for sample pair (xi, xj), where xi is related to the minority class.
6. Repeat the previous step until achieving the minimally Euclidean distanced neighbors for the sample pair (x_i_, x_j_) that is called Tomek-link.
7. Exclusion of x related to the majority class from the Tomek link. (End of Tomek)

**Figure 1:**
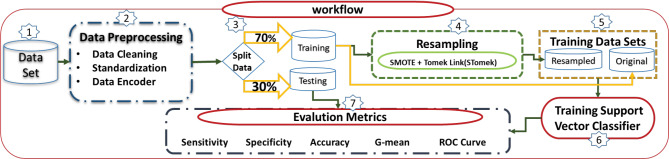
Flowchart of fitted steps in Models 2 and 3 with the difference that Model 2 does not include step 4.

Models 2 and 3 were implemented in Python programing language (version 3.7) using the scikit-learn package.^24^Figure 1 depicts the steps of fitting Models 2 and 3, with the difference that step 4, which is related to data balancing (resampling method), is not implemented in Model 2 but Model 3.

**Figure 2:**
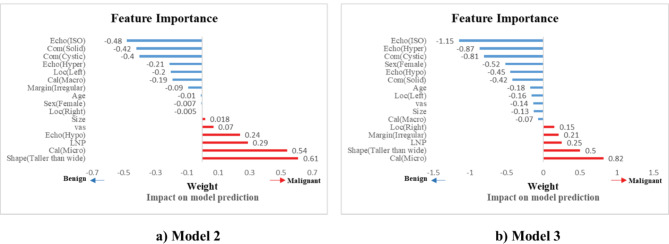
Feature importance for SVC models.

 Moreover, permutation-importance function from the Scikit-learn package^24^ was utilized to elicit weights of important variables in predicting Models 2 and 3 (shown in Figure 2). In this Figure, to distinguish between factors effective in predicting malignancy and benignity of thyroid nodules and for variables effective in predicting malignancy (positive class), weight is marked with a positive sign. On the other hand, for variables effective in predicting benign nodules, the weight is considered with a negative sign.

 Five measures of sensitivity, specificity, accuracy, area under the curve (AUC), and geometric mean (Gmean) were used to evaluate the models. Gmean- an index that balances the model's performance in the two majority and minority classes- is defined as follows^25^:


$$Gmean=\sqrt{Sensitivity\times Specificity}$$

## Results

**Figure 3:**
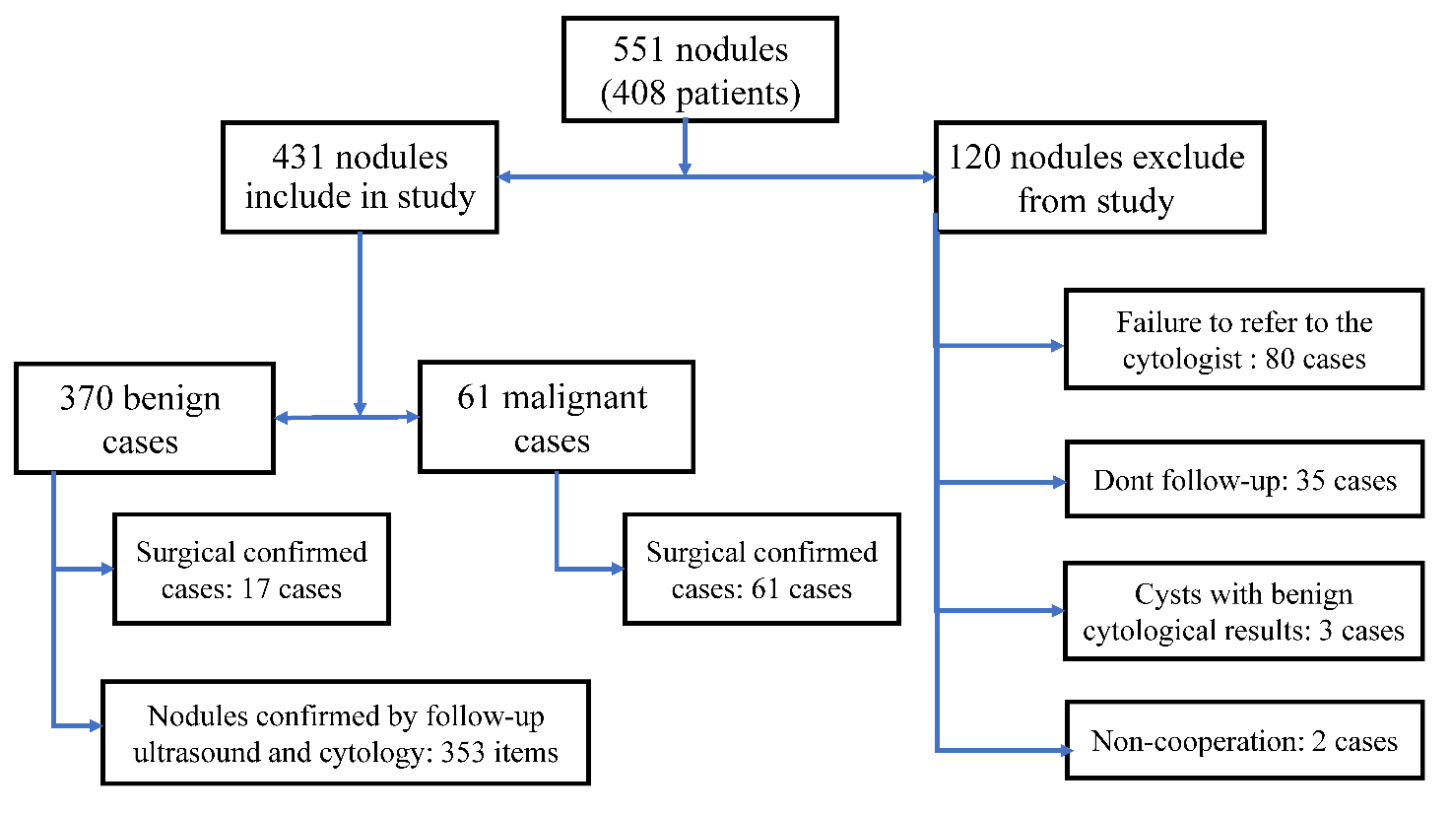
Patients included in the study.

 In this study, 551 nodules out of 408 patients were examined for inclusion in the study, of which 120 nodules were excluded from the study (Figure 3). Finally, 431 nodules out of 313 patients were included in the study. Furthermore, the prevalence of malignant nodules was 14% (n=61). The mean ages of patients with benign and malignant nodules were 48 and 40 years, respectively. Moreover, 87% of the patients were women; however, there was no difference in the prevalence of malignancy between genders. Since the *P* value of the Kolmogorov-Smirnov test violated the normal distribution (*P*<0.05) for variables of age and nodule size (response variable), the Mann-Whitney nonparametric test was used to investigate their relationship with nodule type. In addition, the chi-square test was utilized for the association between qualitative variables and nodule type (Table 1).

**Figure 4:**
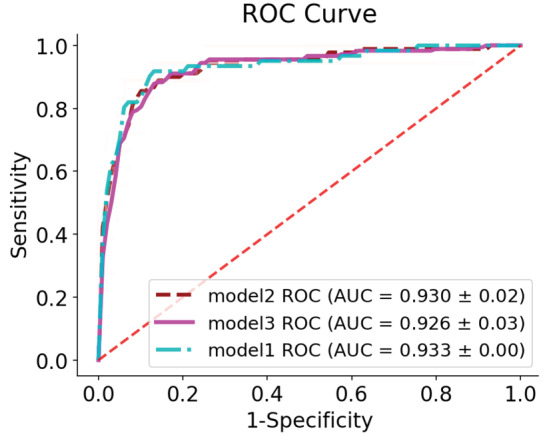
ROC Curve for study models.

*Model 1*: This model included multiple LR model classification thyroid nodules with 60.6% sensitivity and 97.2% specificity. The accuracy and Gmean in this model were 92.1% and 76.8%, respectively. The ROC curve for this model is shown in Figure 4, and the AUC for this model was 93±0.02%. The variables of age, echogenicity (ISO class), calcification (Micro class), nodule shape (Taller than wide class), and nodules with vascularity were statistically significant (0.034, <0.001, <0.001, 0.001, and 0.036, respectively). The odds ratio (OR) for variables was shown in Table 1.

*Model 2*: In this model, the power for predicting malignant nodules sensitivity (63.3%), predictive power of benign nodules (specificity, 95.9%), overall model accuracy (91.3%), and value of Gmean (77.6%) were obtained. You can also see the ROC curve for this prediction model for five random repetitions in Figure 4. The AUC index for this model was 93±0.03%. The important variables in the prediction for this model are plotted in Figure 2. According to this chart, the existence of variables, nodule shape (taller than wide category), calcification (micro class) and in contrast, the absence of echogenicity variables (ISO and hyper classes) and composition (cystic), as well as the most effective variables in the diagnosis of thyroid nodule malignancy were identified.

*Model 3*: Sensitivity and specificity for this model were obtained at 76.1% and 93.8%, respectively. Furthermore, the model's efficiency in terms of accuracy, Gmean, and AUC were equal to 91.3%, 84.2%, and 92.6%, respectively. The ROC curve for this model is drawn in Figure 4. The important variables of the prediction in this model are plotted in Figure 2. According to this chart, the existence of variables, calcification (micro class), nodule shape (Taller than wide category), and in contrast, the absence of echogenicity variables (ISO and hyper classes) and composition(cystic), as well as the most effective variables in the diagnosis of thyroid nodule malignancy were identified.

Table 2 shows the values of the evaluation indicators with a 95% confidence interval for all three models in the study.

**Table 2:** The common evaluation indicators with a 95% confidence

**Evaluation metrics**	**Model І**		**Model П**		**Model Ш**	
	**Scores**	**95% CI**	**Scores**	**95% CI**	**Scores**	**95% CI**
Sensitivity	60.6	48.0, 73.0	63.3	53.3, 73.4	76.1	69.2, 83.0
Specificity	97.2	96.0, 99.0	95.9	94.2, 97.6	93.8	91.8, 95.9
Accuracy	92.1	89.4, 94.2	91.3	89.4, 93.4	91.0	89.8, 93.0
Area under the curve	93.2	92.7, 94.8	93.0	91.3, 94.7	92.6	91.0, 94.2
Gmean	76.8	70.2, 84.6	77.6	71.3, 83.9	84.2	80.7, 87.7

## Discussion

 The prevalence of malignant nodules in this study was obtained at 14%. The mean ages of patients with benign and malignant nodules were 48 and 40 years, respectively, which had a statistically significant difference. This study attempted to show the existing challenges and their effectiveness on statistical models' performance in classifying thyroid nodules, provide a solution for them, and develop a statistical model based on machine learning for screening thyroid nodules.

 Accuracy, AUC, and Gmean were utilized to evaluate the overall performance of models. Accuracy and AUC were almost similar, with the superior performance of Model 1 over the other two models. While according to Gmean, Model 1 shows the weakest performance and Model 3 offers the best performance. Now the question is whether the performance comparison of models should be based on which of the mentioned evaluation metrics?

 According to practical and theoretical evidence, accuracy in imbalanced data is substantially skewed. When the bulk of data in a binary classification is negative, a shallow learning model can achieve high accuracy by classifying the negative class while having poor prediction for the positive class.^26,27^ As a result, using accuracy to evaluate models appears to be essentially worthless in our analysis.

 AUC is a widely used assessment indicator for classification models that is calculated by measuring the area under the ROC curve. This index indicates the difference between true and false positives. Its value, however, is reliant on the ROC curve's threshold (each distinct threshold point generates a different value of the paired values (TP, FP). It will be ideal value when the optimal threshold point is found and established. Otherwise, the index will be biased when evaluating models fitted by imbalanced data.^25,28-31^ It is critical to understand that one method for determining the best threshold for ROC curve is to utilize the Gmean.^26^

 The Gmean is the correct answer because, as previously stated, this index indicates the model's ability to predict both positive (malignancy) and negative (benign) classes to the greatest extent possible balance. A low Gmean value implies that the classification model is heavily skewed toward one class and not the other.^25,28,32,33^ Although Gmean minimizes the negative impact of skewed class distributions, it neither discerns the contribution of each class to the overall performance nor is it the dominant class. Different sensitivity (true positive rate) and specificity (true negative rate) combinations may produce the same result for those two metrics. Therefore, to check the performance of the models, it is necessary to use separate indicators for each class, such as sensitivity and specificity, along with overall measures for both classes.

 To clarify this issue, we can compare the value of the three metrics against the difference between the sensitivity and specificity for each model. Sensitivity and specificity for Model 1 were equal to 60.6% and 97.2% (difference: 36.6%), for Model 2 were equal to 63.3% and 95.9% (difference: 32.6%), and for Model 3 were equal to 76.1% and 93.8% (difference: 17.7%). The difference between the first two models is considerably greater than that in Model 3. This difference is typically more visible when the data used to build the model contains imbalanced classes, causing the model to bias toward the majority class (benign nodules). Since the value of specificity in these two models is substantially greater than the value of sensitivity, the value of accuracy and AUC metrics in these models is bigger than the value of the Gmean. These metrics are created in such a manner that they cannot be a good indicator of the model's ability to predict both classes,^27^ but the Gmean has overcome this issue and has been able to demonstrate the model's sensitivity and specificity concurrently.^34^ Meanwhile, unlike Models 2 and 3, cross-validation-training and testing process were not used to evaluate Model 1. It was traditionally fitted and assessed with a single dataset, which could cause over fitting in the results of this model.^23^ However, Models 2 and 3 have been evaluated in 5 replications using the test dataset. Finally, Model 3 was chosen as the top model based on the Gmean and the higher sensitivity than the other two models when comparing the models in overall performance (predictive power of both classes) as a consequence of the considerations above.

 Most thyroid nodules are benign, and the imbalance data in this topic appears to be evident. However, a few researchers have focused on aspects listed in predicting malignant thyroid nodules. For example, Chen et al, Ouyang et al, and Prochazka et al all used machine learning algorithms to classify thyroid nodules.^6,7,35^ To evaluate the models, they have only reported the AUC or accuracy index and have not even reported the sensitivity and specificity. In contrast to the three studies mentioned, Ma et al utilized the Gmean index to report model performance in their research to identify thyroid nodules using SVM. In their study, the Gmean index, sensitivity, and specificity were found to be 90%, 93.8%, and 86.6%, respectively.^36^ Although their study data had imbalanced classes, it was not conducted to balance the data, as we did in our analysis.

 Based on the best model in this study, we chose the most important variables in classifying thyroid nodules (Figure 2). Micro calcification is one of the categories of calcification, which is the most important predictor in the diagnosis of thyroid nodule malignancy based on the selected model. This feature is considered the second effective factor in diagnosing malignancy according to Model 2, and according to Model 1 is one of the features that has a significant effect on the prediction of malignant thyroid nodules.

 Taller than wide shape: This feature was the second most effective predictor of malignancy in terms of the selected model, the best predictor of malignancy in Model 2, and one of the influential variables in Model 1. In some sources, Taller than wide shape has been introduced as the best predictor for malignant nodules.

 Lymphadenopathy: This characteristic was likewise established as one of the influential factors in the diagnosis of malignancy for all three models. However, due to the small number of samples having this feature in the research (7 samples), we skipped incorporating it in among the influential variables. Irregular speculated or micro lobulated margin has also proven effective in malignancy in Model 3. In all three models, ISO and hyperechogenicity play a key role in identifying benign nodules for classification. In some research, ISO echogenicity has been introduced as the best predictor for predicting benignity. Based on Models 3 and 2, having a predominantly cystic feature is also a sign of benign thyroid nodules. Taller than wide shape, micro calcifications, and irregular margins were reported as the most practical characteristics in predicting thyroid nodule malignancy in many investigations, including the meta-analysis by Remonti et al.^34,37-40^

 However, like most other research, this one includes limitations that might bias the findings. Due to a lack of resources, time, and access to a large data bank, ultrasound images could not be employed directly in this model. If this was feasible, we could deploy image processing to allow the model to extract hidden characteristics from the radiologist and use them to enhance the model's performance.

**Highlights**

- The prevalence of malignant nodules is obtained at 14%.
- The SVC using the Smote-Tomek algorithm to balance the training dataset showed the best performance.
- Unbalanced data caused the models to be misdirected towards the majority class.
- Accuracy evaluation criteria and area under the curve without using optimal point provided misleading results for models, while the geometric mean was not like this.
- The micro calcification, taller than wide shape, as well as lack of ISO and hyperechogenicity features were identified as the most effective malignant variables.

## Conclusion

 Our study results clearly show the trained model's increased sensitivity using balanced data, compared to the unbalanced and traditional prediction methods. It is possible to construct a pre-FNA screening system for thyroid nodules classification by addressing the described flaws and providing acceptable solutions to data challenges, particularly class imbalances in this field. In addition, saving time and treatment costs, as well as patient stress can be achieved due to its indirect effects.

## Acknowledgements

 This study was extracted from a research project approved by Babol University of Medical Sciences and Health Services, Babol, Iran (IR.MUBABOL.REC.1398.034). The authors would like to express their gratitude to the Research Center of Babol University of Medical Sciences, as well as all dear ones who helped the researchers in conducting this study.

## Conflict of interest

 There is no conflict of interest.

## Funding

 No funding.
